# Supplementary material for: Venomix: a simple bioinformatic pipeline for identifying and characterizing toxin gene candidates from transcriptomic data
Source: PeerJ. 2018 Jul 31;6:e5361. doi: 10.7717/peerj.5361 (PMC6074769; doi:10.7717/peerj.5361)
Supplement: Supplemental Information 6 [file peerj-06-5361-s006.gz › FinalOutput_1E-6/Phospholipase_A2_1/finaltree.pdf]

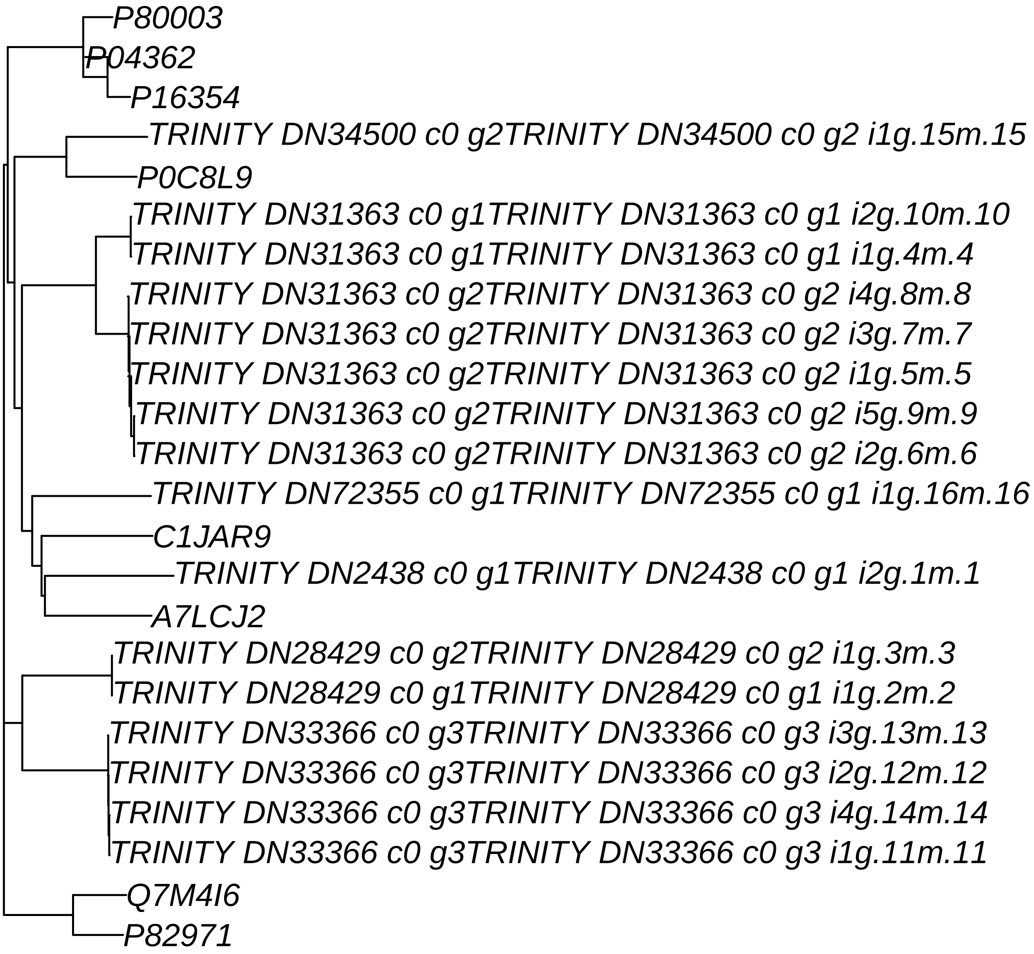

P80003
P04362
P16354
TRINITY DN34500 c0 g2TRINITY DN34500 c0 g2 i1g.15m.15
P0C8L9
TRINITY DN31363 c0 g1TRINITY DN31363 c0 g1 i2g.10m.10
TRINITY DN31363 c0 g1TRINITY DN31363 c0 g1 i1g.4m.4
TRINITY DN31363 c0 g2TRINITY DN31363 c0 g2 i4g.8m.8
TRINITY DN31363 c0 g2TRINITY DN31363 c0 g2 i3g.7m.7
TRINITY DN31363 c0 g2TRINITY DN31363 c0 g2 i1g.5m.5
TRINITY DN31363 c0 g2TRINITY DN31363 c0 g2 i5g.9m.9
TRINITY DN31363 c0 g2TRINITY DN31363 c0 g2 i2g.6m.6
TRINITY DN72355 c0 g1TRINITY DN72355 c0 g1 i1g.16m.16
C1JAR9
TRINITY DN2438 c0 g1TRINITY DN2438 c0 g1 i2g.1m.1
A7LCJ2
TRINITY DN28429 c0 g2TRINITY DN28429 c0 g2 i1g.3m.3
TRINITY DN28429 c0 g1TRINITY DN28429 c0 g1 i1g.2m.2
TRINITY DN33366 c0 g3TRINITY DN33366 c0 g3 i3g.13m.13
TRINITY DN33366 c0 g3TRINITY DN33366 c0 g3 i2g.12m.12
TRINITY DN33366 c0 g3TRINITY DN33366 c0 g3 i4g.14m.14
TRINITY DN33366 c0 g3TRINITY DN33366 c0 g3 i1g.11m.11
Q7M4I6
P82971
